# Latent Factor Decoding of Multi-Channel EEG for Emotion Recognition Through Autoencoder-Like Neural Networks

**DOI:** 10.3389/fnins.2020.00087

**Published:** 2020-03-02

**Authors:** Xiang Li, Zhigang Zhao, Dawei Song, Yazhou Zhang, Jingshan Pan, Lu Wu, Jidong Huo, Chunyang Niu, Di Wang

**Affiliations:** ^1^Key Laboratory of Medical Artificial Intelligence, Shandong Computer Science Center (National Supercomputer Center in Jinan), Qilu University of Technology (Shandong Academy of Sciences), Jinan, China; ^2^School of Computer Science and Technology, Beijing Institute of Technology, Beijing, China; ^3^Software Engineering College, Zhengzhou University of Light Industry, Zhengzhou, China

**Keywords:** latent factor decoding, emotion recognition, EEG, deep learning, variational autoencoder

## Abstract

Robust cross-subject emotion recognition based on multichannel EEG has always been hard work. In this work, we hypothesize that there exist default brain variables across subjects in emotional processes. Hence, the states of the latent variables that relate to emotional processing must contribute to building robust recognition models. Specifically, we propose to utilize an unsupervised deep generative model (e.g., variational autoencoder) to determine the latent factors from the multichannel EEG. Through a sequence modeling method, we examine the emotion recognition performance based on the learnt latent factors. The performance of the proposed methodology is verified on two public datasets (DEAP and SEED) and compared with traditional matrix factorization-based (ICA) and autoencoder-based approaches. Experimental results demonstrate that autoencoder-like neural networks are suitable for unsupervised EEG modeling, and our proposed emotion recognition framework achieves an inspiring performance. As far as we know, it is the first work that introduces variational autoencoder into multichannel EEG decoding for emotion recognition. We think the approach proposed in this work is not only feasible in emotion recognition but also promising in diagnosing depression, Alzheimer's disease, mild cognitive impairment, etc., whose specific latent processes may be altered or aberrant compared with the normal healthy control.

## 1. Introduction

In recent years, affective computing has started to become an active research topic in fields of pattern recognition, signal processing, cognitive neuropsychology, etc. Its main objective is exploring effective computer-aided approaches in recognizing a person's emotions automatically by utilizing explicit or implicit body information, e.g., through facial expressions or voices. It has wide application prospects within the field of human-computer interaction (e.g., intelligent assistants and computer games) (O'Regan, 2003; Moshfeghi, 2012) and psychological health care (Sourina et al., 2012) the WHO estimates that depression, as an emotional disorder, will soon be the second leading cause of the global burden of disease (WHO, 2017).

Considering that facial or vocal muscle activity can be deliberately controlled or suppressed, researchers are currently starting to explore this question through implicit neural activities, particularly through the multichannel EEG (electroencephalograph). The neural oscillations revealed by the EEG are highly correlated with various dynamic cognitive processes (Ward, 2004), including the emotional processes. Hence, its multichannel monitoring and high temporal resolution provide us with possibilities in exploring robust indicators and computational methods for EEG-based emotion recognition.

Nevertheless, there exist some major problems with regards to multichannel EEG-based emotion recognition that need to deal with, such as the poor generalization of data across subjects and the limitations in designing and extracting handcrafted emotion-related EEG features. Further, in medical data-mining tasks, acquiring enough manually labeled data for training supervised models remains a problem. How to fully utilize the limited data to enhance the model performance is worthy of exploration. Hence, unsupervised and handcrafted featured non-dependent modeling methods are worth in-depth exploration.

In this work, we have utilized the findings of prior related works (Adolphs et al., 1994; Vytal and Hamann, 2014) and raised the hypothesis that, though differences exist between individuals, there exist also intrinsic default variables (e.g., brain networks or intracranial current sources) that take part in emotional processes. Then, the characteristics of these intrinsic variables can be utilized for analyzing different emotional states. Specifically, in this work, three unsupervised autoencoder-like neural network models have been utilized to model the multichannel EEGs and infer the state space of the latent factors. Based on the state sequences of the factors, the participants' emotional status can be estimated by applying a contextual modeling method. According to the experimental results, the unsupervised neural network models are effective and feasible in modeling multichannel EEG, and the inferred factors indeed contain emotion-related information that are beneficial for further emotion recognition.

## 2. Materials and Methods

### 2.1. An Overview of the Framework

Emotional processes are higher-order cognitive processes that are produced by the collaborative involvement of various latent brain factors, including different brain areas and physical or functional brain networks. The status information of the latent factors contains emotion-related information that contribute to estimating the emotional status. Hence, how to effectively and precisely infer the latent factors is the core issue that we have been concerned with in this work. As the EEG is the external manifestation of the latent brain factors' activities, the recorded EEGs of different scalp locations having internal associations, it has provided us with a way to infer the latent factors from the external multichannel EEGs.

**Figure 1:**
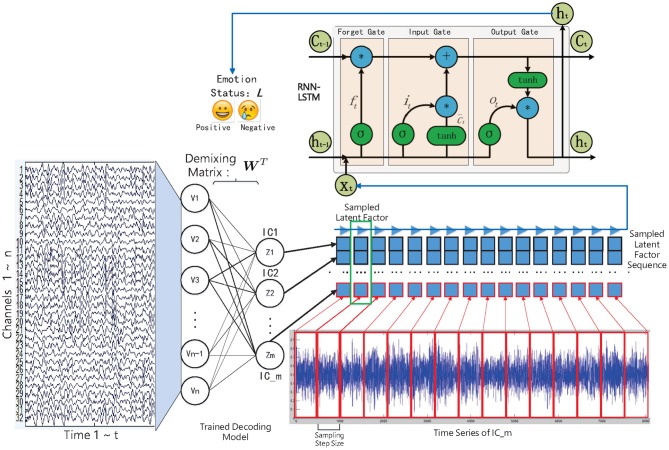
The decoded EEG factors and recurrent neural network-based emotion recognition approach framework.

In this work, we have studied and compared three kinds of autoencoder-form neural network models, including the traditional autoencoder (AE), the variational autoencoder (VAE), and the restricted Boltzmann machine (RBM) to determine the latent factors from the multichannel EEG data. Furthermore, for estimating the emotional status, after training, the state sequences of the latent factors were modeled by contextual learning models (e.g., the LSTM unit-based recurrent neural network); at the same time, the emotional status can be estimated based on the contextual information. The entire method framework is illustrated in the flow chart as in Figure 1.

### 2.2. Neural Network-Based Latent Factor Decoding Models

Latent factor decoding from a brain activity signal is a key tool for studying cognitive task performance and impairment (Calhoun and Adali, 2012). The decoded factors can be further utilized to locate the intracranial current sources or identify intrinsic brain networks. Most of the popular methods for inferring latent factors have the core assumption of the existence of hidden factors that are mixed to produce the observed data (Calhoun et al., 2010).

Traditionally, in order to model latent factors, we first need to determine the independent components (ICs) from the multichannel brain signals by solving a blind source separation (BSS)/single matrix factorization (SMF) problem, among which the independent component analysis (ICA) is the most commonly used method (Chen et al., 2013). Specifically, the multichannel EEGs are expressed as a channels-by-time data matrix ***E***^*n*×*t*^, where the *t* is the number of measured time points of a signal, and the *n* is the number of electrodes (channels). Solving the BSS/SMF problem is to discover the underling latent source factors ***S***^*m*×*t*^ from the external multichannel EEG signals, where the *m* is the number of hypothesized factors, and *t* is the number of data points in one source signal. The relationship between the multichannel EEGs and the latent source factors is expressed in Formula 1:

(1) $$\begin{array}{r} E^{n\times t}\approx M^{n\times m}S^{m\times t}, \end{array}$$

where the channels-by-sources matrix ***M*** is the unknown “mixing” matrix. Hence, for determining the latent factors, we need to find the “demixing” matrix ***D***, which is the inverse of the matrix ***M*** that satisfies ***S*** ≈ ***DE***.

In the above latent factor decoding studies, the “demixing” matrix ***D*** and the ICs are determined through some methods based on matrix factorization (e.g., the ICA). However, the utilization of ICA has been limited by its flexibility and representation ability (Choudrey, 2002). Hence, its effectiveness in the scenario of cross-subject decoding and recognition is questionable. Currently, various deep learning (DL) models have been applied to solve supervised or unsupervised learning problems in fields of computer vision and natural language processing. Besides, DL-based approaches can learn intermediate concepts, which could yield better transfer across source and target domains (Glorot et al., 2011). Recent works have verified that the fMRI volume-based DL approach can identify comparable latent factors to the ICA-based approach (Huang et al., 2016). Hence, this inspires us to introduce neural network models to solve the problem of latent factor decoding from emotional EEG data.

#### 2.2.1. Traditional Autoencoder-Based Decoding Approach

The basic autoencoder model is a feedforward neural network that consists of symmetrical network structures: the “encoder” and “decoder.” To be more specific, consider one dataset $X={\left\{x^{\left(t\right)}\right\}}_{t=1}^{N}$ of variable *x*. As in Formula 2, the encoder is responsible for encoding the input into a higher-level (and generally compressed) representation, which we call the “bottleneck.” Then, as in Formula 3, the decoder is responsible for reconstructing the input data based on the hidden representation. The parameters of the AE are optimized by minimizing the difference (reconstruction error) between the output data and input data, as in Formula 4.

(2) $$\begin{array}{rcl} h_{j}^{\left(t\right)} & = & f\left(\underset{i}{\sum }W_{ij}^{1}x_{i}^{\left(t\right)}+b_{1}\right) \end{array}$$

(3) $$\begin{array}{r} \bar{x_{i}^{\left(t\right)}}=f\left(\underset{j}{\sum }W_{ij}^{2}h_{j}^{\left(t\right)}+b_{2}\right) \end{array}$$

(4) $$\begin{array}{r} L\left(W^{1},W^{2},b_{1},b_{2};X\right)=\underset{x^{\left(t\right)}\in X}{\sum }∥x^{\left(t\right)}-\bar{x^{\left(t\right)}}∥ \end{array}$$

The AE is generally trained through a back propagation (BP) method. As we can see, the AE shares some practical similarities with the SMF models. To some extent, the weight matrices ***W***_1_ and ***W***_2_ can also been regarded as “demixing” and “mixing” secret keys, respectively. Then, the relationship between the observed EEGs and the latent factors can be determined by them.

#### 2.2.2. Restricted Boltzmann Machine-Based Decoding Approach

A restricted Boltzmann machine (RBM) is a kind of undirected probabilistic graph model with no connections between units of the same layer. It provides the possibility of constructing and training deeper neural networks (Hinton and Salakhutdinov, 2006). From a probabilistic modeling perspective, the latent factors learned in an RBM give a description of the distribution over the observed data. Specifically, an RBM specifies the distribution over the joint space [*x, h*] via the Boltzmann distribution, as in Formula 5:

(5) $$\begin{array}{r} p\left(x,h;\theta \right)=\frac{1}{Z_{\theta }}exp\left(-\varepsilon_{\theta }^{RBM}\left(x,h\right)\right) \end{array}$$

in which *Z*_θ_ is the normalization term, and $\varepsilon_{\theta }^{RBM}\left(x,h\right)$ is the system energy function, namely:

(6) $$\begin{array}{r} \varepsilon_{\theta }^{RBM}\left(x,h\right)=-x^{T}Wh-a^{T}x-b^{T}h \end{array}$$

where θ = {*W, a, b*} are the model parameters that respectively encode the visible-to-hidden interactions (W), the visible self-connections (a), and the hidden self-connections (b). The visible and hidden nodes of RBM are typically binary statistic units. Nevertheless, for EEG data, the visible nodes need to model a distribution that is an approximately real value and Gaussian. Hence, the RBM adopted here is the Gaussian RBM, where the conditional distribution of a single hidden and visible node is given by:

(7) $$\begin{array}{r} P\left(h_{j}=1|x\right)=\sigma \left(\underset{i}{\sum }W_{ij}x_{i}+b_{j}\right) \end{array}$$

and

(8) $$\begin{array}{r} x_{i}\sim N\left(\sigma_{i}\underset{i}{\sum }W_{ij}h_{j}+a_{i},\sigma_{i}\right) \end{array}$$

where σ(.) is the logistic function and N(μ,σ) is the normal distribution with mean μ and standard deviation σ. Further, we make a corresponding modification for the energy function, as in Formula 9:

(9) $$\begin{array}{r} \varepsilon_{\theta }^{RBM}\left(x,h\right)=-\underset{ij}{\sum }\frac{x_{i}}{\sigma_{i}}W_{ij}h_{j}-\underset{i}{\sum }\frac{{\left(a_{i}-x_{i}\right)}^{2}}{\sigma^{2}}-\underset{j}{\sum }b_{j}h_{j} \end{array}$$

The parameters θ = {*W, a, b*} are optimized by training the RBM to maximize the likelihood of the observed data: $\overset{N}{\underset{t=1}{\sum }}P\left(x^{\left(t\right)};\theta \right)$. The traditional gradient descent-based method to maximize the likelihood is intractable in the RBM based approach. This problem is solved by approximating the gradient through Markov Chain Monte Carlo (MCMC) where contrastive divergence (CD) with truncated Gibbs sampling is applied to improve computational efficiency (Hinton, 2002). The model is further unrolled to a symmetrical auto-encoder structure, whose parameters, as was discovered in the CD process, are fine-tuned with a back-propagation (BP) process, much like the traditional auto-encoders.

#### 2.2.3. Variational Autoencoder-Based Decoding Approach

Very recently, the variational autoencoder (VAE) was introduced as a powerful DL model for some problem scenarios that needed modeling of the data's probability distribution (Kingma and Welling, 2014). The objective function of traditional AE only measures the value difference between the input and output vector. The difference in distribution cannot be reflected and controlled. Compared with the traditional AE model, the VAE model provides a closed-form representation of the distribution underling the input data, which is quite suitable for unsupervised learning of the latent factors.

It hypothesizes that all the data are generated by one random process that involves an unobservable latent variable *z*. The latent variable is generated from one prior distribution *p*_θ_(*z*), and the *x* is determined by the conditional distribution *p*_θ_(*x*|*z*). Both the parameters θ and the latent variable *z* are unknown to us. The direct inference of the latent variable *p*_θ_(*z*|*x*) is intractable. Hence, in the design of the VAE, one recognition model *q*_ϕ_(*z*|*x*) is introduced to approximate the true posterior *p*_θ_(*z*|*x*). The VAE utilizes the probabilistic encoder structure to encode the input into latent variables (*q*_ϕ_(*z*|*x*)), and it further utilizes the probabilistic decoder structure to map the latent variables to reconstructed input (*p*_θ_(*x*|*z*)). The optimization objective of the VAE is expressed in Formula 10:

(10) $$\begin{array}{r} \begin{array}{c} \text{max}{𝔼}_{q_{\phi }\left(z|x\right)}\left[\text{log}p_{\theta }\left(x|z\right)\right]-D_{KL}\left(q_{\phi }\left(z|x\right)||p_{\theta }\left(z\right)\right) \end{array} \end{array}$$

This is also referred to as the variational lower bound.

The first term 𝔼_*q*_ϕ_(*z*|*x*)_[log*p*_θ_(*x*|*z*)] is the expectation of the logarithmic likelihood with regard to the approximate posterior *q*_ϕ_(*z*|*x*). It can be obtained through Monte Carlo estimate, namely through sampling *L* times, as in Formula 11:

(11) $$\begin{array}{r} \frac{1}{L}\overset{L}{\underset{l=1}{\sum }}\text{log}p_{\theta }\left(x^{\left(t\right)}|z_{l}^{\left(t\right)}\right) \end{array}$$

The second term −*D*_*KL*_(*q*_ϕ_(*z*|*x*)||*p*_θ_(*z*)) is the KL divergence of the approximate posterior *q*_ϕ_(*z*|*x*) from the true prior *p*_θ_(*z*). It is computed through Formula 12:

(12) $$\begin{array}{r} \frac{1}{2}\overset{J}{\underset{j=1}{\sum }}\left(1+\text{log}\left(\sigma_{j}^{\left(t\right)2}\right)-\mu_{j}^{\left(t\right)2}-\sigma_{j}^{\left(t\right)2}\right) \end{array}$$

Let J be the dimensionality of *z*.

**Figure 2:**
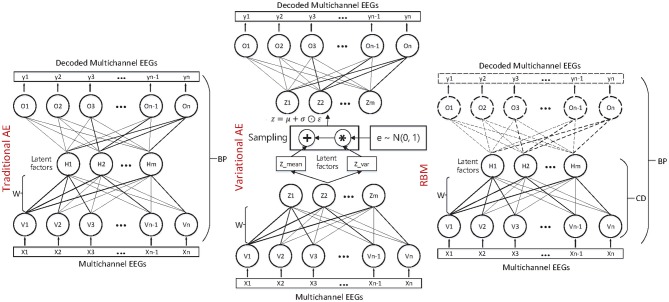
The neural network-based multichannel EEG fusion and latent factor decoding method.

To sum up, the prior variational lower bound can be further transformed into the following form in Formula 13:

(13) $$\begin{array}{c} \begin{array}{c} L\left(\theta ,\phi ;x^{\left(t\right)}\right)≃\frac{1}{2}\overset{J}{\underset{j=1}{\sum }}\left(1+\text{log}\left(\sigma_{j}^{\left(t\right)2}\right)-\mu_{j}^{\left(t\right)2}-\sigma_{j}^{\left(t\right)2}\right)\\ +\frac{1}{L}\overset{L}{\underset{l=1}{\sum }}\text{log}p_{\theta }\left(x^{\left(t\right)}|z_{l}^{\left(t\right)}\right)\; \end{array} \end{array}$$

where the *z* is sampled with a reparameterization trick, namely $z_{l}^{\left(t\right)}=\mu^{\left(t\right)}+\sigma^{\left(t\right)}⊙\epsilon_{l}$ and $\epsilon_{l}\sim N\left(0,I\right)$, and the ⊙ refers to the element-wise product. Both the *q*_ϕ_(*z*|*x*) and *p*_θ_(*z*) are assumed to obey the centered isotropic multivariate Gaussian, namely $q_{\phi }\left(z|x^{\left(t\right)}\right)=N\left(z;\mu^{\left(t\right)},\sigma^{\left(t\right)2}I\right)$, where the mean μ^(*t*)^ and standard deviation σ^(*t*)^ are the computation outputs of the encoder network with respect to the input *x*^(*t*)^ and the variational parameter ϕ. The VAE is trained through stochastic gradient descent and back-propagation (BP) method. Compared with the traditional matrix factorization-based approach, the VAE is formulated as a density estimation problem. The structure and mechanism of the three autoencoder-like neural network models are illustrated in Figure 2.

### 2.3. Contextual Learning From Latent Factors for Emotion Recognition

According to some studies, the generation of the emotional experience generally lags behind the activity of the brain neural systems (Krumhansl, 1997). The recurrent neural network (RNN), meanwhile, has the ability to accumulate useful information at each time step, by which the influence of the lag-effect can be eliminated. This is important when we do not know which moment plays the most important role in the subject's final evaluation of the specific emotion they experienced in a trial. In view of this, we have considered adopting the RNN model to perform sequence modeling on the decoded latent factor sequences; meanwhile, the subjects' emotional status can be estimated, as shown in Figure 1, whereas traditional RNN's practical application is limited by the “gradient vanish” in back-propagation when its dependencies is too long. Some rectified recurrent units have been adopted in the RNN model, in which the LSTM unit that contains a “gate” structure has gained great success in various sequence-modeling tasks, such as speech recognition (Graves et al., 2013). The popular LSTM unit-based RNN model was therefore selected in this work.

Specifically, in this work, we have fed the multichannel EEGs into the “encoder” part of the trained models to obtain the corresponding latent factor sequences, namely the independent components (ICs): $\mathrm{IC}={\left\{IC^{\left(t\right)}\right\}}_{t=1}^{N}$. The high sampling rate EEG signal also corresponds to the high sampling rate ICs, which will lead to the high computational cost in sequence modeling. Here we need sampling from the entire ICs to construct samples for LSTM training. For the *m* sampled elements from the entire ICs, the mechanism of the recognition model can be formulated as:

(14) $$\begin{array}{r} <IC^{\left(1\right)},\ldots ,IC^{\left(t\right)},\ldots ,IC^{\left(m\right)}>\mapsto L^{\left(m\right)}\mapsto L \end{array}$$

which follows the “many-to-one” mode.

### 2.4. Experimental Dataset

We examined the proposed approach on two publicly accessible datasets, including **DEAP** (Koelstra et al., 2011) and **SEED** (Zheng and Lu, 2015). DEAP included 32-channel EEG data collected from 32 subjects, and the subjects rated their emotional experience on a two-dimensional emotional scale, namely Arousal (which ranges from relaxed to aroused) and Valence (which ranges from unpleasant to pleasant). The higher the specific rating was, the more intense the emotion was in a specific dimension. SEED included 62-channel EEG data collected from 15 subjects. After data acquisition, some basic preprocessing processes were conducted, such as removing the electrooculogram (EOG) and electromyogram (EMG) artifacts.

The samples of DEAP were divided into positive and negative samples according to the ratings on the Valence and Arousal emotional dimensions. A sample with score over five points was considered to be a positive class, while a sample with a score below five points was considered a negative class. The SEED dataset had pre-defined negative and positive emotional classes for the samples that we did not need to conduct label processing.

## 3. Results and Discussion

### 3.1. EEG Decoding Method Settings

In addition to removing EEG artifacts, we conducted z-score method-based normalization for each subject's channel data. For comparison, we built a one-hidden-layer structure for the neural network models, and the number of hidden nodes was set according to the number of latent factors we set in advance (DEAP: 2–16, SEED: 2–31). Both of the two datasets were acquired with high sampling rate. Take the DEAP dataset for example; the number of samples of one subject was over 320,000. Hence, considering the training speed, we set the batch size for unsupervised latent factor learning as 500. The loss functions were selected and set according to the descriptions in section 2.2. We selected the Adam and RMSprop method for AE and VAE training, respectively. According to the experiments, the loss function can converge to the minimum within 20 training epoches. The RBM model-based approach was realized through the Matlab DeeBNet V3.0 deep belief network toolbox, whereas the AE- and VAE-based approaches were realized through the deep learning framework–Keras based on Tensorflow backend. More experimental setting details can be accessed in the source codes located in the following repository: https://github.com/muzixiang/LatentFactorDecodingEEG.

For comparison, we also set an experiment of an ICA-based decoding approach, and selected FastICA as the implementation method, which is most widely used and accepted in the resolution of EEG source localization and blind source separation problems. This is due to the fact that the ICA-based approach has problems in determining the specific order of the latent components as well as the reconstructed multi-channel EEGs. In this work, we also took this problem into consideration. Specifically, for each original channel EEG, we measured the correlation between it and each reconstructed signal. We supposed that the reconstructed signal with the highest correlation was the counterpart of the specific original EEG, and the highest correlation was adopted here to measure the reconstructed performance of the ICA-based approach. Besides, the reconstruction experiment and performance measurement were conducted 10 times to increase the reliability of the results, and the average performance was reported in this paper. Though we know this is a crude approach, and there may be some mistakes in determining the counterpart for the original EEG, the reported results here were indeed the best estimation and constituted the performed upper bound for ICA.

Besides, the performance of the PCA-based approach was also presented. Nevertheless, the PCA and ICA were totally different in theory and application scenarios. The PCA-based approach was generally used as a dimension reduction method, which is not to mine the underling random processing but try to extract the most important information that can best represent the original data. In this work, we were interested in the EEG latent factors-based approaches. Anyway, as a classic method, the PCA was also worth exploring, and we also added experiments when the PCA approach was adopted.

### 3.2. Emotion Recognition Method Settings

Although LSTM unit-based RNN has the ability to process long-term sequences, in the case of high-sampling rate EEG signals, signal sequences with hundreds of thousands of data points can introduce significant time overhead in sequence learning. Hence, as shown in Figure 1, the training sequences were constructed based on the sampling step size, and the data were sampled from the sequence at equal intervals according to the step size. This strategy was good for quickly verifying the experimental results. In the experiment, we set the sampling step size as 0.25 s. The number of input layer nodes of the LSTM unit were determined by the number of latent factors. The number of output hidden layer nodes was set to 200, and the output nodes were fully connected with one hidden layer containing 100 Relu-type nodes. At the end of the model, a decision-making layer with Softmax-type nodes that represent different emotional state was connected. Besides, the Dropout operation was set for the last two fully connected layers. The model loss function was set to binary cross entropy, the batch size was set to 50, and the RMSprop algorithm was selected as the optimization method.

We also set baseline methods based on handcrafted features for comparison with our framework. We choose the support vector machine (SVM) combined with the L1-norm penalty-based feature selection method (SVM-L1). Besides, random forests (RF), K-nearest neighbors (KNN), logistic regression (LR), naive bayes (NB), and the feed-forward deep neural network (DNN) were also examined. As listed in Table 1, three main categories of EEG features were extracted for Theta rhythm, Alpha rhythm, Beta rhythm, and Gamma rhythm, including nine kinds of time-frequency domain features (TFD features), nine kinds of non-linear dynamical system features (NDS features), and 14 pairs of brain hemisphere asynchronous activity features (BHAA features). Hence, For the DEAP dataset, the total number of feature dimensions for one trial was 2360 (4_*rhythms* × 32_*channels* × (9_*TFfeatures*+9_*NDSfeatures*)+4_*rhythms* × 14_*BHAAfeatures*). For the SEED dataset, the total number of features extracted for one trial was 4520 (4_*rhythms* × 62_*channels* × (9_*TFfeatures*+9_*NDSfeatures*)+4_*rhythms* × 14_*BHAAfeatures*). Besides, several related representative works in recent years are also compared.

**Table 1:** Three main categories of EEG features that we extracted for baseline methods.

**Feature Type**	**Extracted Features**
Time-frequency	1. Peak-Peak Mean. 2. Mean Square Value.
domain features	3. Variance. 4. Hjorth Parameter:Activity.
	5. Hjorth Parameter: Mobility.
	6. Hjorth Parameter: Complexity.
	7. Maximum Power Spectral Frequency.
	8. Maximum Power Spectral Density.
	9. Power Sum.
Non-linear dynamical	1. Approximate Entropy. 2. C0 Complexity.
system features	3. Correlation Dimension.
	4. Kolmogorov Entropy.
	5. Lyapunov Exponent.
	6. Permutation Entropy. 7. Singular Entropy.
	8. Shannon Entropy. 9. Spectral Entropy.
Asynchronous brain	1. Fp1-Fp2. 2. AF3-AF4. 3. F3-F4.
activity features	4. F7-F8. 5. FC5-FC6. 6. FC1-FC2.
	7. C3-C4. 8. T7-T8. 9. CP5-CP6.
	10. CP1-CP2. 11. P3-P4. 12. P7-P8.
	13. PO3-PO4. 14. O1-O2.

### 3.3. Evaluation Metrics

For evaluating the reconstruction performance, we adopted the Pearson correlation coefficient as the metric to measure the difference between the input original channel signal and the output reconstructed signal, as in Formula 15. In other words, high r-value indicated the model has good ability in reconstructing the time series. This metric gave us a general view of the feasibility and effectiveness of the model in modeling the multichannel EEG data.

(15) $$\begin{array}{r} r_{x_{in}x_{out}}=\frac{\sum_{t=1}^{n}\left(x_{in}^{\left(t\right)}-\bar{x_{in}}\right)\left(x_{out}^{\left(t\right)}-\bar{x_{out}}\right)}{\sqrt{\sum_{t=1}^{n}{\left(x_{in}^{\left(t\right)}-\bar{x_{in}}\right)}^{2}}\sqrt{\sum_{t=1}^{n}{\left(x_{out}^{\left(t\right)}-\bar{x_{out}}\right)}^{2}}} \end{array}$$

For evaluating the emotion recognition performance, we chose to leave one subject's data out of the cross-validation method to compare our framework with the baseline methods. Every time, we left one subject's data out as the test set and adopt the other subjects' data as the training set. Considering the problem of unbalanced classes, the model performance was evaluated on the test set based on the F1-score metric, as in Formula 16. This procedure iterates until each subject's data has been tested.

(16) $$\begin{array}{r} P_{f1}=2\cdot \frac{Precision\cdot Recall}{Precision+Recall} \end{array}$$

### 3.4. Evaluation on EEG Modeling and Reconstruction

**Figure 3:**
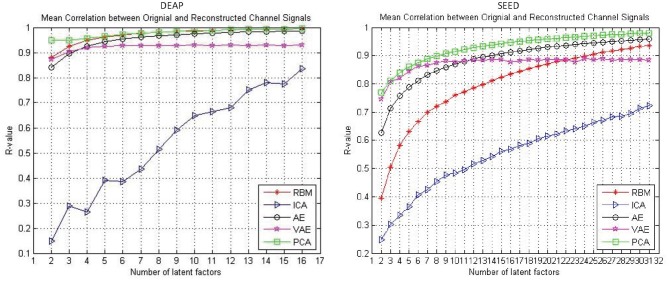
The reconstruction performance (mean Pearson correlation coefficient) of different decoding models when assuming a different number of latent factors.

The reconstruction performance under different assumed number of latent factors was of interest. In this work, we examined the reconstruction performance with varying number of latent factors. Specifically, considering the experimental cost, we only examined the number of latent factors from two to half the number of EEG channels. As shown in Figure 3, the performance was gradually improved with the increased number of latent factors for all the approaches. It can be found that, with the increase of the number of latent factors, the AE and RBM even obtained an approximate 100% reconstruction performance on the DEAP dataset. Nevertheless, it should be noted that the VAE-based modeling method was special, and its reconstruction performance did not always improve with the increase of the number of latent factors. The mean correlation coefficient gradually stabilized at around 0.9. Besides, when tested on the SEED dataset, the VAE-based method could achieve a better reconstruction performance than other methods with fewer hidden layer node settings, which indicated that the method had the ability to mine the most important latent factors from multichannel EEGs. The PCA performed well on both datasets, as expected; however, the PCA-based approach was a kind of dimension reduction method, which was not to mine the underling random process. We still need to examine their effectiveness in recognizing subjects' emotions by further utilizing the pattern recognition methods.

**Figure 4:**
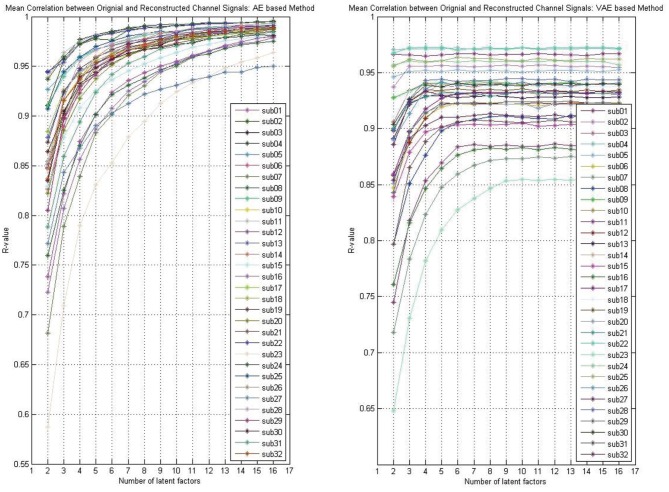
The reconstruction performance (mean Pearson correlation coefficient) of different subjects when assuming a different number of latent factors (take the DEAP for example).

Whether the reconstruction performance was consistent across subjects when assuming different latent factors is worth exploring. As shown in Figure 4, we presented the AE model- and VAE model-based reconstruction performance with varying number of latent factors on each subject's data. The experimental results indicated that, for each subject's data, the performance improves gradually with the increase of the number of latent factors, and we obtain relatively smooth curves on each of the subjects'data. Though, for the VAE model, there was a little fluctuation, the performance on all subjects' data eventually stabilized.

As shown in Figure 3, the reconstruction performance of the ICA-based method was always much lower than the neural network-based modeling method. This suggested that neural network based approaches are more suitable for modeling and decoding brain neural signals than ICA based method. According to the Universal Approximation Theorem, a neural network structure with a single hidden layer can approximate any function. In other words, even if we restrict our networks to have just a single layer intermediate between the input and the output neurons–a so-called single-hidden-layer network–such a simple network architecture can be extremely powerful. Hence, it is not surprising that the neural networks adopted in this work can achieve a good reconstruction performance. As illustrated in Figure 4, the neural network obtained a relatively stable reconstruction performance on each subject's data. The performance increased gradually and achieved a sufficiently good performance when setting proper number of latent factors. It also indicated the effectiveness and robustness of the neural networks in decoding and reconstructing multichannel EEGs.

**Figure 5:**
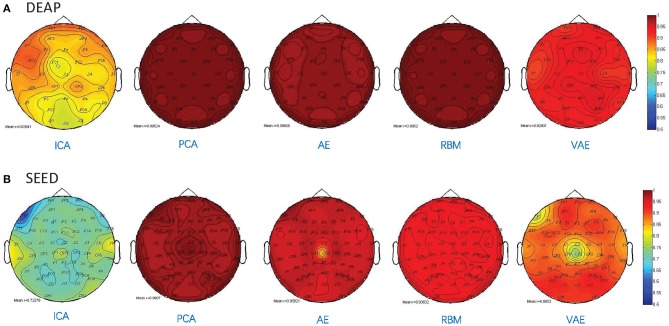
Pearson correlation coefficient between the reconstructed EEG signal and the original EEG signal for each channel. **(A)** The reconstruction performance of different methods on DEAP dataset. **(B)** The reconstruction performance of different methods on SEED dataset.

Besides, as shown in Figure 5, we illustrated the performance of each method in reconstructing the multichannel EEGs through a channel layout heatmap format. The mean Pearson correlation coefficient over all the subjects is presented. The greater the value, the darker the color. Specifically, the figure shows the reconstructed performance when the number of latent factors is set as half the number of electrode channels (DEAP: 16 latent factors, SEED: 31 latent factors), which achieve the best reconstruction performance, as shown in Figure 3, and the averaged r-values are presented. It can be seen that the AE- and RBM-based methods on both datasets achieved the best reconstruction effect. Nevertheless, the frequently used ICA-based method was obviously inferior to other methods on the whole, and there existed significant performance imbalance in different brain regions. It indicated that the ICA-based EEG modeling approach was not robust compared to the neural network-based approaches.

### 3.5. Evaluation on Latent Factor-Based Emotion Recognition

As mentioned above, the performance of each unsupervised modeling method on EEG reconstruction cannot be used as a criterion for judging whether the model successfully deciphers latent factors that contribute to emotion recognition. It is necessary to apply pattern recognition methods on those mined factors, and conduct a comparison based on the recognition performance. Specifically, the LSTM takes charge of modeling the latent factor sequence decoded by the ICA-, AE-, RBM-, and VAE-based approaches and also inferred the emotional state. Besides, the performance, when applying the LSTM to the principle components mined by the PCA method, has also been reported.

The classification performance is evaluated when the number of the latent factor is set as half of the number of total electrodes. Namely, for the DEAP dataset, the number of latent factors used for sequence modeling and classification was 16, whereas, for the SEED dataset, the number of latent factors was set as 31. We think the emotion recognition performance must closely related to the EEG reconstruction performance. In other words, the latent factors with low reconstruction performance were not an accurate reflection of the latent EEG process and could not lead to an ideal emotion classification result. Hence, the emotion recognition experiments on both datasets were conducted on the latent factors with the high reconstruction performance, namely 16 and 31. Besides, the datasets were recorded with very high sampling rate; take the DEAP dataset for example, the total number of EEG samples of just one subject was over 320,000 the experimental cost for training, evaluating the models, and storing the decoded latent factors are high. Evaluating on more parameter settings is somewhat impractical in our current experimental conditions, e.g., for the SEED dataset, a total of 5 methods × 62 factors × 15 subjects = 4,650 different experimental settings were needed. Furthermore, the purpose of this work was to verify the effectiveness of the EEG latent factor-based emotion recognition method and was not to find the best parameter settings; the reconstruction performance achieved when the number of latent factor was half of the number of electrodes was good enough to test our idea.

We adopted a “leave one subject's data out” cross-validation method. For the DEAP dataset, Tables 2, 3 summarize the recognition performance on the emotional dimension of Valence and Arousal, respectively. Table 4 summarizes the recognition performance on SEED dataset. Considering the problem of unbalanced classes, the recognition performance was measured and compared with each other using the F1 score.

**Table 2:** Recognition performance on subject data of DEAP dataset (Valence).

**Subject No**.	**ICA+LSTM**	**PCA+LSTM**	**AE+LSTM**	**RBM+LSTM**	**VAE+LSTM**
s01	0.5818	0.6296	0.6207	0.6296	0.6552
s02	0.5882	0.6897	0.6885	0.7018	0.7213
s03	0.7097	0.6897	0.7241	0.7097	0.7097
s04	0.4444	0.5455	0.5490	0.5000	0.5818
s05	0.5965	0.7000	0.7302	0.7500	0.7541
s06	0.7241	0.7500	0.7419	0.8529	0.8182
s07	0.7368	0.7619	0.7586	0.8065	0.8125
s08	0.6071	0.6415	0.6545	0.6780	0.7213
s09	0.5217	0.4898	0.5926	0.6545	0.6552
s10	0.6207	0.6182	0.6441	0.6441	0.7059
s11	0.6909	0.6552	0.7097	0.7419	0.7500
s12	0.6667	0.6909	0.6552	0.6885	0.7000
s13	0.4186	0.5714	0.6182	0.5882	0.6182
s14	0.4444	0.5926	0.6182	0.6429	0.6780
s15	0.5200	0.6143	0.6667	0.6441	0.6667
s16	0.4706	0.5217	0.5306	0.5385	0.5660
s17	0.6786	0.6552	0.6885	0.6897	0.7097
s18	0.6667	0.6983	0.7407	0.7213	0.7500
s19	0.7059	0.6697	0.7302	0.7302	0.7419
s20	0.7018	0.7119	0.7097	0.7302	0.7719
s21	0.6667	0.6600	0.6441	0.6780	0.6667
s22	0.5306	0.5333	0.5965	0.6207	0.6207
s23	0.7000	0.7170	0.7119	0.7241	0.8000
s24	0.5106	0.5714	0.6207	0.5714	0.6316
s25	0.4889	0.5532	0.6441	0.6441	0.6441
s26	0.6415	0.7619	0.7333	0.7692	0.7813
s27	0.8060	0.8309	0.8125	0.8235	0.8529
s28	0.6786	0.7070	0.7241	0.7692	0.7619
s29	0.6667	0.6667	0.7097	0.7143	0.7541
s30	0.7742	0.7241	0.7813	0.8125	0.8182
s31	0.6667	0.6897	0.7018	0.7302	0.7213
s32	0.6441	0.5769	0.6538	0.6667	0.6897
Mean *P*_*f*1_	0.6303	0.6528	0.6783	0.6927	0.7167

*^1^number of latent factors: 16*.

**Table 3:** Recognition performance on subject data of DEAP dataset (Arousal).

**Subject No**.	**ICA+LSTM**	**PCA+LSTM**	**AE+LSTM**	**RBM+LSTM**	**VAE+LSTM**
s01	0.6545	0.7241	0.7419	0.7419	0.7500
s02	0.7119	0.7018	0.7458	0.7500	0.7619
s03	0.2791	0.3182	0.3043	0.3111	0.3478
s04	0.4138	0.5714	0.5106	0.5660	0.5714
s05	0.5600	0.6441	0.6316	0.6316	0.6441
s06	0.5200	0.6182	0.5818	0.5818	0.6275
s07	0.7213	0.7338	0.7541	0.7500	0.7869
s08	0.6667	0.6667	0.7213	0.7018	0.7333
s09	0.6885	0.7302	0.7419	0.7213	0.7636
s10	0.5660	0.6154	0.6667	0.6667	0.7097
s11	0.5217	0.4898	0.5455	0.5306	0.5556
s12	0.8000	0.8732	0.8889	0.8889	0.9041
s13	0.5385	0.7125	0.7419	0.9041	0.8889
s14	0.7241	0.7385	0.7619	0.7937	0.8060
s15	0.5882	0.6316	0.6545	0.6552	0.6667
s16	0.6273	0.6296	0.6545	0.6667	0.6667
s17	0.6667	0.7302	0.7241	0.7333	0.7869
s18	0.7541	0.7500	0.7692	0.7500	0.7742
s19	0.7213	0.7213	0.8060	0.7097	0.8065
s20	0.7879	0.8060	0.8235	0.8657	0.8732
s21	0.8529	0.8406	0.8732	0.8696	0.9014
s22	0.7241	0.7458	0.7500	0.7500	0.7500
s23	0.3673	0.3404	0.3750	0.4000	0.4000
s24	0.8358	0.8889	0.8696	0.8732	0.9041
s25	0.7500	0.7458	0.8182	0.8406	0.8406
s26	0.5714	0.5769	0.5769	0.5965	0.6071
s27	0.6780	0.7797	0.8060	0.7879	0.8060
s28	0.6071	0.6071	0.6154	0.6207	0.6545
s29	0.7018	0.7000	0.7458	0.7419	0.7586
s30	0.6182	0.5769	0.6207	0.6207	0.6441
s31	0.5769	0.6182	0.6316	0.6316	0.6552
s32	0.7213	0.7419	0.7692	0.7541	0.8148
Mean *P*_*f*1_	0.6418	0.6741	0.6944	0.7002	0.7243

*^1^number of latent factors: 16*.

**Table 4:** Recognition performance on subject data of SEED dataset.

**Subject No**.	**ICA+LSTM**	**PCA+LSTM**	**AE+LSTM**	**RBM+LSTM**	**VAE+LSTM**
s01	0.6753	0.7195	0.7368	0.7677	0.8308
s02	0.6897	0.7636	0.7386	0.7721	0.8504
s03	0.7255	0.7259	0.7674	0.8018	0.8741
s04	0.6434	0.6495	0.6677	0.6915	0.7012
s05	0.6683	0.7221	0.7323	0.7552	0.8027
s06	0.7321	0.7333	0.7733	0.8054	0.8904
s07	0.7143	0.7713	0.7525	0.7953	0.8600
s08	0.7053	0.7479	0.7488	0.7897	0.8571
s09	0.6912	0.7080	0.7430	0.7759	0.8538
s10	0.6677	0.7599	0.7264	0.7452	0.7931
s11	0.7295	0.7548	0.7696	0.8026	0.8827
s12	0.6776	0.7095	0.7373	0.7718	0.8320
s13	0.7168	0.7894	0.7560	0.7962	0.8676
s14	0.6939	0.7586	0.7442	0.7876	0.8565
s15	0.7608	0.7699	0.7844	0.8079	0.8908
Mean *P*_*f*1_	0.6994	0.7389	0.7452	0.7777	0.8429

*^1^number of latent factors: 31*.

It is found from the table that the ICA-LSTM-based method on both datasets exceeds 0.5, indicating that the traditional ICA-based method can still decipher emotion-related information from the multichannel EEG. It also indicates that the latent factor decoding combined with sequence modeling-based approach is suitable for emotion recognition from multichannel EEG. In general, the ICA-based approach did not perform as well as other neural network-based approaches, confirming the conclusion that ICA has limitations in representation ability (Choudrey, 2002). The RBM- and VAE-based approaches are better than the ICA- and AE-based approaches. This indicates that the generative models are more suitable in the current scenario.

(17) $$\begin{array}{r} \underset{\omega_{0},\omega }{\min}\overset{n}{\underset{i=1}{\sum }}\left[1-y_{i}\left(\omega_{0}+\overset{q}{\underset{j=1}{\sum }}\omega_{j}x_{i,j}\right)\right]+C||\omega |{|}_{1} \end{array}$$

**Figure 6:**
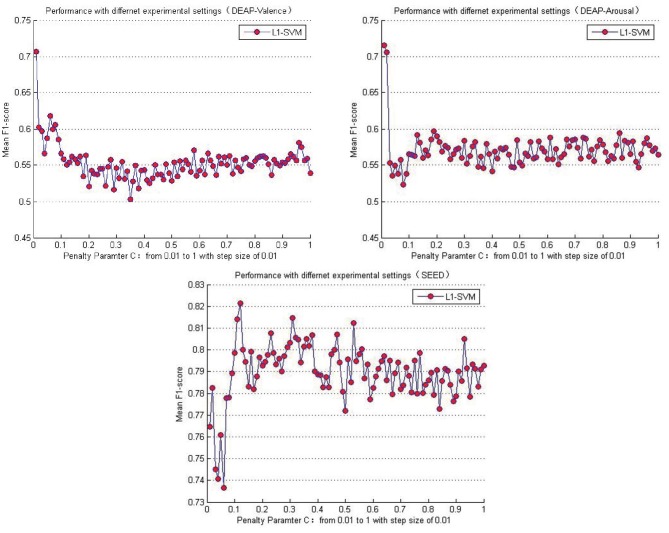
Mean cross-subject recognition performance with different settings when L1-SVM based approach is applied.

Table 5 lists the performance comparison between the baseline methods and the proposed approaches in this paper. Among the several baseline methods, the SVM combined with the L1-norm penalty-based feature selection method (L1-SVM) achieved the best performance when applying the optimum parameter. As shown in Formula 17, this method introduced the L1-norm regularization term ||ω||_1_ into the objective function to induce sparsity by shrinking the weights toward zero. It is natural for features with 0 weights to be eliminated from the candidate set. The parameter “C” controls the trade-off between the loss and penalty. Hence, the results of the performance when a different penalty parameter “C” was tested are shown in Figure 6. Then the best performances on DEAP and SEED datasets are reported in Table 5.

**Table 5:** Performance comparison between this work and the baseline methods.

**Approach/Model**	**Performance (P**_**f1**_**)**		
	**DEAP**		**SEED**
	**Valence**	**Arousal**	
L1-SVM (kernel=“linear”) + handcrafted features	0.7134	0.7154	0.8234
RF (n_estimators=100) + handcrafted features	0.5870	0.5754	0.7680
KNN (n_neighbors=7) + handcrafted features	0.6406	0.5890	0.6763
LR + handcrafted features	0.5757	0.5614	0.7649
NB + handcrafted features	0.6903	0.6989	0.6666
DNN (hidden_layer_sizes={100, 100, 100}) + handcrafted features	0.6183	0.6593	0.7219
ICA+LSTM	0.6303	0.6418	0.6994
PCA+LSTM	0.6528	0.6741	0.7389
AE+LSTM	0.6783	0.6944	0.7452
RBM+LSTM	0.6927	0.7002	0.7777
VAE+LSTM	0.7167	0.7243	0.8429

Compared to the baseline methods, the VAE-based approach achieved higher performance on both datasets. It should be pointed out that, though the performance shown here was not good enough compared with the L1-SVM method, it avoided the problems of high computational cost when calculating the handcraft features, especially for the Non-linear Dynamical System Features (e.g., the Lyapunov exponent). Besides, the effectiveness of the features highly depends on the parameter settings (e.g., the setting of the number of the embedding dimension when calculating the Lyapunov exponent). When extracting the features from multichannel EEGs, the cost will multiply. This issue hampers the practical usage of the EEG-based emotion recognition. Hence, compared to the traditional handcraft feature-based methods, the proposed neural network-based approach was advantageous in terms of data-processing speed when the trained network was provided in advance. The process of latent factor decoding, sequence modeling, and classification can be conducted and completed at a very fast speed. In addition, the experimental settings and parameters of our approach were not fully tested. On the whole, the approach proposed in this paper is also valuable and has great potential in this field. The AE has shown excellent ability in reconstructing the multichannel EEG; however, according to the recognition performance, its decoded factors were not an accurate reflection of the brain cognitive state compared to the RBM- and VAE-based approaches. Hence, in cognition research-oriented neural signal computing, the generative model-based approaches are more advisable. Finally, despite the excellent performance the PCA obtained in reconstructing the multichannel EEGs, the principle components mined by it do not contribute to promoting the performance in recognizing subjects' emotions.

As shown in Table 6, we furthermore list the highly cited related works in recent years and the corresponding performance obtained. Though the performance obtained in this work was slightly inferior to some related works, it verified the effectiveness of the proposed approach and inspires us to do further research, such as finding the best model parameters and studying the brain functions based on the decoded latent factors.

**Table 6:** List of related works in recent years and the corresponding performance obtained.

	**Approach/Model**	**Performance**			
		**Valence**		**Arousal**	
		***P*_*acc*_**	***P*_*f*1_**	***P*_*acc*_**	***P*_*f*1_**
DEAP
	Ontology-based storage and representation, feature selection and decision tree based recognition method (Chen et al., 2015)	0.6783	N/A	0.6896	N/A
	Minimum-redundancy-maximum-relevance (MRMR) based feature selection combined with the statistical features, band power features, Hjorth parameters and fractal dimension (Atkinson and Campos, 2016)	0.7314	N/A	0.7306	N/A
	Integrated classifier based on multi-layer stacking autoencoder combined with time domain features and PSD features (Yin et al., 2017)	0.7617	0.7243	0.7719	0.6901
	Multivariate empirical mode decomposition (MEMD) based feature extraction combined with ANN (Mert and Akan, 2018)	0.7287	N/A	0.7500	N/A
	Generative adversarial network (WGANDA) based transfer learning combined with differential entropy feature (Luo et al., 2018)	0.6799	N/A	0.6685	N/A
	The VAE based approach proposed in this work	0.7623	0.7167	0.7989	0.7243
		***P***_***acc***_		***P***_***f*1**_	
SEED
	Dynamical graph CNN (DGCNN) learns from the DE, PSD, DASM, RASM and DCAU features based adjacency matrix representation (Song et al., 2018)	0.7995		N/A	
	Extracting differential entropy features to construct 2D sparse graph representation, then combining CNN for classification (Li et al., 2018)	0.8820		N/A	
	Transfer learning methods combined with differential entropy features and logistic regression based classification (Lan et al., 2018)	0.7247		N/A	
	Generative adversarial network (WGANDA) based transfer learning combined with differential entropy feature (Luo et al., 2018)	0.8707		N/A	
	Spatial-temporal recurrent neural network (STRNN) combined with differential entropy feature (Zhang et al., 2018)	0.8950		N/A	
	The VAE based approach proposed in this work	0.8581		0.8429	

## 4. Conclusion

This paper explored EEG-based emotion identification methods that were not restricted to handcrafted features. Brain cognition research finds that “there exists cross-user, default intra-brain variables involved in the emotional process.” Hence, the status of the brain hidden variables is closely related with the emotional psychophysiological processes and can be utilized to infer the emotional state. In this work, artificial neural networks are used for unsupervised modeling of the state space of the latent factors from the multichannel EEGs, and LSTM-based supervised sequence modeling is further performed on the decoded latent factor sequences to mine the emotion related information, which is used for inferring the emotional states. It has been verified that the neural network models are more suitable for modeling and decoding brain neural signals than the independent component analysis (ICA) method, which is widely used in brain cognitive research. Although, from the perspective of data reconstruction, the VAE cannot achieve the same performance as that of the traditional AE, we obtained a better recognition performance on the latent factors decoded by the VAE. It indicated that VAE, as a kind of generative model, can truly model the hidden state space of the brain in cognitive processes. The decoded latent factors contain the relevant and effective information for emotional state inference. This approach is also promising in diagnosing depression, Alzheimer's disease, mild cognitive impairment, etc., whose specific brain functional networks may have been altered or could be aberrant compared with the normal healthy control. In future work, we will study the influence of a different sampling step size in latent factor sequence modeling and emotion recognition. Other directions deserving of exploration in future works include source localization and functional network analysis based on the decoded latent factors.

## Data Availability Statement

The DEAP dataset analyzed for this study can be found in the following link: http://www.eecs.qmul.ac.uk/mmv/datasets/deap/index.html. The SEED dataset analyzed for this study can be found in the following link: http://bcmi.sjtu.edu.cn/~seed/index.html.

## Author Contributions

XL proposed the idea, realized the proposed approach, conducted experiments and wrote the manuscript. ZZ provided advice on the research approaches, guided the experiments, checked, polished the manuscript and provided the experimental environment. DS proposed the idea, reviewed the related works, analyzed the results and polished the manuscript. YZ realized the proposed methods, conducted experiments, analyzed the results and provided revision suggestions. JP and LW analyzed the experimental results, checked this work and provided revision suggestions. JH conducted experiments of the baseline methods for comparison and summarized the results. CN and DW acquired, pre-processed the experimental dataset and extracted the handcraft EEG features.

### Conflict of Interest

The authors declare that the research was conducted in the absence of any commercial or financial relationships that could be construed as a potential conflict of interest.
